# Microbiome Research and Multi-Omics Integration for Personalized Medicine in Asthma

**DOI:** 10.3390/jpm11121299

**Published:** 2021-12-05

**Authors:** Marianthi Logotheti, Panagiotis Agioutantis, Paraskevi Katsaounou, Heleni Loutrari

**Affiliations:** 1 G.P. Livanos and M. Simou Laboratories, 1st Department of Critical Care Medicine & Pulmonary Services, Evangelismos Hospital, Medical School, National Kapodistrian University of Athens, 3 Ploutarchou Str., 10675 Athens, Greece; marianthilogotheti@gmail.com (M.L.); panagiout@med.uoa.gr (P.A.); 2 Biotechnology Laboratory, School of Chemical Engineering, National Technical University of Athens, 5 Iroon Polytechniou Str., Zografou Campus, 15780 Athens, Greece; 3 Pulmonary Dept First ICU, Evangelismos Hospital, Medical School, National Kapodistrian University of Athens, Ipsilantou 45-7, 10675 Athens, Greece; paraskevikatsaounou@gmail.com

**Keywords:** asthma, gut and airway microbiota, systems biology, multi-omics data integration, bioinformatics, biomarkers, drug targets, precision medicine

## Abstract

Asthma is a multifactorial inflammatory disorder of the respiratory system characterized by high diversity in clinical manifestations, underlying pathological mechanisms and response to treatment. It is generally established that human microbiota plays an essential role in shaping a healthy immune response, while its perturbation can cause chronic inflammation related to a wide range of diseases, including asthma. Systems biology approaches encompassing microbiome analysis can offer valuable platforms towards a global understanding of asthma complexity and improving patients’ classification, status monitoring and therapeutic choices. In the present review, we summarize recent studies exploring the contribution of microbiota dysbiosis to asthma pathogenesis and heterogeneity in the context of asthma phenotypes–endotypes and administered medication. We subsequently focus on emerging efforts to gain deeper insights into microbiota–host interactions driving asthma complexity by integrating microbiome and host multi-omics data. One of the most prominent achievements of these research efforts is the association of refractory neutrophilic asthma with certain microbial signatures, including predominant pathogenic bacterial taxa (such as *Proteobacteria* phyla, *Gammaproteobacteria* class, especially species from *Haemophilus* and *Moraxella* genera). Overall, despite existing challenges, large-scale multi-omics endeavors may provide promising biomarkers and therapeutic targets for future development of novel microbe-based personalized strategies for diagnosis, prevention and/or treatment of uncontrollable asthma.

## 1. Introduction

Asthma is a chronic inflammatory disease with multiple phenotypes that causes immune and respiratory dysfunction and globally affects more than 300 million people, leading to nearly half a million deaths [1]. Numerous studies within the last decade have explored the multiple levels of asthma complexity and underlined the concept that asthma rather represents an umbrella term covering distinct phenotypes and pathophysiological mechanisms [2,3,4].

Disease initiation and subsequent exacerbations have been connected to several factors, including an individual’s genetic susceptibility as well as exposure to environmental stimuli and behavioral attributes, such as pathogenic microorganisms, allergens, air pollution, tobacco smoke and diet [5]. Asthma symptoms vary broadly in severity, from wheezing and shortness of breath to cough and chest tightness, as well as airflow obstruction. Regarding management, asthma has been confronted for many years with a universal therapy with bronchodilators and inhaled corticosteroids (ICS), aiming to improve bronchoconstriction and airway inflammation, yet not taking into account the diversity of the pathogenic background underlying distinct disease subtypes. As a result, a wide spectrum of responses to treatment has been observed, while a significant fraction of patients suffering from severe symptoms remains under-treated, causing a substantial burden to individuals, families and health care systems [5,6].

The concept of precision medicine ensuring that each patient is provided, in a timely manner, with the appropriate prognosis, diagnosis and/or therapy, thus eliminating the negative consequences and resulting in the maximum clinical benefit, is clearly relevant to the heterogeneous nature of asthma [7,8,9,10]. Actually, research exploiting modern systems biology approaches that combine individuals’ pathophysiological traits with high-throughput profiling of molecular biomarkers in large, well-characterized cohorts of asthmatics have significantly expanded our ability to better understand asthma complexity, as well as to develop more targeted strategies to disease diagnosis, therapy and monitoring [11,12]. In this same context, a substantial effort has focused on the investigation of patients’ microbiota in the gut and upper/lower airways and its potential connection with the different asthma subtypes [13].

Microbiota is the complex and dynamic community of microbes (bacteria, archaea, viruses and eukaryotic microbes), both commensals and pathogens, that reside in and on a host organism (human, animal, plant) and/or the environment. Microbiome mainly refers to the genome representing the microbiota, yet both terms are often used interchangeably [14]. Human microbiota colonizes shortly after birth in the gastrointestinal and respiratory tracts as well as in other multiple body sites, and from that point on is continuously shaped by various environmental exposures and potentially host genetic background. It is now widely accepted that these microbial communities, along with their metabolites, support many vital biological processes in the host organism (regulation of the immune system, metabolism, brain function, response to drugs, etc.), contributing either to health or disease under eubiosis or dysbiosis conditions, respectively. In fact, dysbiosis, i.e., the imbalance of microbial diversity between beneficial and harmful pathogenic microbes or simply the disturbance of the “normal” abundance of certain commensal microorganisms, has been associated with numerous human disorders, including asthma. However, whether the observed dysbiosis is primary or secondary to disease development remains a subject of debate in most cases [13].

From a technological point of view, the rapid development of next-generation sequencing platforms, which have made sequencing much faster and cheaper, along with the continuous advances of bioinformatics for large dataset analysis and integration, helped to overcome the limitations of conventional microbiology methods and largely promoted unprecedented advances in microbiome research and potential applications [15,16]. In terms of sequencing approaches, amplicon sequencing that targets the bacterial 16S ribosomal RNA (rRNA) gene has been the most widely used, offering the main body of available knowledge regarding the composition and dynamics of bacterial communities. On the other hand, shotgun sequencing of the whole metagenome, although significantly more expensive, with high demands in analytical skills and quite challenging, especially with low-biomass microbial communities, is lately gaining in popularity since it can capture the entire microbial genomic content (bacteria, viruses and eukaryotic microorganisms) and provide a complete taxonomic and functional characterization of the whole microbiota [17].

In this review, we first summarize recent works exploring asthma heterogeneity, especially at a comprehensive -omics analysis level. We then attempt to cover current advances on host microbiome research, particularly considering potential correlations linking the structure of microbial communities with asthma heterogeneous phenotypes/endotypes and treatment. Subsequently, emphasis is given on studies integrating microbiome and host multi-omics data using bioinformatics tools, mathematical models and machine learning approaches. We finally discuss challenges to process, analyze, interpret and combine such big biological datasets, as well as future directions towards precision medicine in asthma.

## 2. Asthma Heterogeneity and Classification

Asthma was initially categorized in two main phenotypes, namely atopic or “extrinsic” asthma, related to an allergic reaction upon exposure to environmental inhaled allergen(s), and non-atopic or “intrinsic” asthma. Atopic asthma presents a higher prevalence in childhood, whereas non-atopic asthma occurs mainly in older ages. The chronic inflammation of atopic asthma is caused by an enhanced immune response against common, non-pathogenic, environmental allergens. In contrast, non-atopic asthma is triggered by various non-allergic factors such as viral infections, tobacco smoking, stress, etc. In both types, patients are characterized by a genetic susceptibility [4,5,18].

Additional phenotypic classification for asthma has been mainly based on single variables, such as the age of onset (childhood/early-onset asthma, adult/late-onset asthma, elderly/very-late onset asthma), disease severity (mild, moderate and severe asthma) or responsiveness to a specific treatment. Other common phenotypic variants in asthma are based on the presence of comorbidities, as for example obesity-associated asthma, or on specific irritants such as smoking-associated asthma and asthma induced by ingestion of aspirin. In addition to the large fraction of asthmatics that are responsive to golden standard medication, basically including bronchodilators and ICS, a specific group of patients presents refractory symptoms despite being administered intense and novel therapies [18,19]. The pathological profile of these patients is generally classified as “severe asthma” and represents 10–20% of the total [4,6]. The phenotypic-based classification has not achieved so far to clearly distinguish discrete subtypes of asthmatic patients, and an overlap between the different groups has been constantly observed.

**Figure 1:**
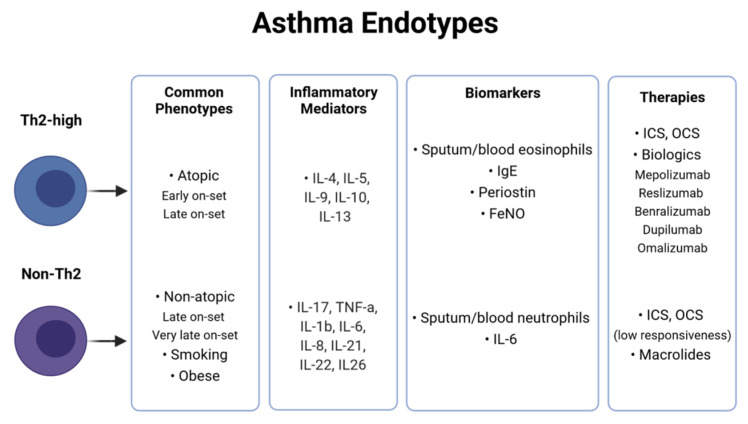
Main features of asthma endotypes. FeNO: fractional exhaled nitric oxide; IgE: immunoglobulin E; IL: interleukin; ICS: inhaled corticosteroids; OCS: oral corticosteroids.

Recently, an alternative arrangement of asthma patients into two major “endotypes”, namely T-helper lymphocytes 2 high (Th2-high) or non-T-helper lymphocytes 2 high (non-Th2), has been suggested based on predominant underlying inflammatory pathways and involved specific biomarkers (Figure 1). The immune-pathogenesis of asthma has been extensively reviewed elsewhere [18,20] and will only be shortly covered here. In brief, T-helper (Th) cells, particularly Th2 and Th17, have been proved to play a central role in asthma: by releasing a number of cytokines and orchestrating a cascade of inflammatory signaling pathways, they can coordinate other immune cells such as B cells, eosinophils, mast cells or neutrophils and ultimately lead to disease pathogenesis [18,21].

In the Th2-high endotype (often referred as eosinophilic asthma), which occurs in approximately 50% of patients with asthma, Th2 cells produce various cytokines, such as interleukins (IL)-4, IL-5, IL-9, IL-10 and IL-13 and induce the recruitment and activation of eosinophils [22]. Among the released cytokines, IL-4 seems to centrally contribute to atopy by stimulating B cells to produce Immunoglobulin E (IgE), which, in turn, boosts mast cells to release histamine, serotonin, and leukotrienes to cause bronchoconstriction and mediate allergic responses. Furthermore, IL-5 exerts pleiotropic effects on eosinophils by promoting their proliferation, maturation, activation, survival and migration to airways. Elevated sputum and blood eosinophils, as well as high levels of total serum IgE, proteins derived from the bronchial epithelium such as serum periostin and fractional exhaled nitric oxide (FeNO), are currently exploited as diagnostic biomarkers indicative of Th2-high type asthma [23,24]. Common asthma phenotypes included in the Th2-high asthma endotype are early-onset atopic asthma and late-onset eosinophilic asthma. The clinical symptoms in Th2-high asthmatics can range from mild to severe, and usually, patients are responsive to ICS standard treatment [4]. To tackle severe, persistent Th2-high inflammation, a more precise approach has been recently applied to design novel biomarker-oriented “biologics”, mainly monoclonal antibodies, such as omalizumab targeting peripheral IgE, mepolizumab, reslizumab and benralizumab targeting IL-5 pathways and finally dupilumab that targets IL-4 and IL-13 pathways [10]. The selection of the appropriate biological treatment strategy depends on various factors, such as the levels of serum IgE or eosinophils. For example, high periostin and IgE serum levels combined with blood eosinophils and elevated FeNO can indicate the response to omalizumab therapy [25]. Sputum and blood levels of eosinophils without IgE can predict the responsiveness to anti-IL-5 treatment [10].

Non-Th2 endotype, or non-eosinophilic asthma, is characterized by a lack of Th2-dependent inflammation. Other Th cell types, mainly Th17, but also Th9, Th25, Th3 and regulatory T cells (Tregs), have been shown to contribute to the inflammatory processes that lead to the initiation or aggravation of asthma. Th17 cells produce potent pro-inflammatory cytokines such as IL-17A, IL-17F, tumor necrosis factor alpha (TNF-α), IL-1b, IL-6, IL-8, IL-21, IL-22 and IL-26 [26,27,28] and coordinate the recruitment of neutrophils in the airways [29]. According to the pattern of airway inflammation, non-Th2 asthma is classified under the following subcategories: neutrophilic, mixed granulocytic and paucigranulocytic. Diagnostically, neutrophilic asthma involves neutrophil cell counts in the sputum within the range of 40–70%; mixed granulocytic inflammation is characterized by the presence of both neutrophils and eosinophils; finally, paucigranulocytic asthma presents normal eosinophils and neutrophils proportions in the sputum. Non-Th2, particularly neutrophilic, asthma is related to non-atopic phenotype, old age, pollutants, viral/microbial infections, tobacco smoking and obesity. Interestingly, it has been shown that neutrophilic asthma can be induced by *Moraxella*, *Streptococcus* and *Haemophilus* species [30]. Non-Th2 asthma is characterized by severe symptoms and poor response to inhaled and oral corticosteroids (OCS) [31]. Levels of sputum neutrophils are considered as a biomarker for predicting symptoms’ persistence despite standard treatment, and it is indicative for macrolides administration in severe asthma [32]. Currently, IL-6 and metalloproteinase 9 (MMP9) are suggested as novel biomarkers for non-Th2 asthma [4].

Based on the Th2-high and non-Th2 asthma classification according to specific inflammatory biomarkers and the emergence of new biologic therapies, the Global Initiative for Asthma (GINA) has developed a Pocket Guide for helping health professionals to identify and decide on the most appropriate treatment strategy that can effectively cure asthma patients, especially those exhibiting persistent Th2-high inflammation [6]. However, the mechanisms driving the refractory non-Th2 subtypes, such as neutrophilic asthma, are still poorly understood and there is not, so far, an effective management for severely ill asthmatics. Besides, the considerable side effects associated with high OCS administration point to a more individualized choice of both the potentially beneficiary patients and the optimum corticosteroid therapeutic doses [33]. These issues have urgently prompted the further pursuit of precision medicine perspectives in modern asthma research using systems biology approaches.

## 3. Insights from Host-Omics Research

During the last two decades, the rapid development of -omics approaches regarding new technologies and novel systems biology computational analyses has inevitably infiltrated asthma research, as clearly documented by relevant literature [34,35,36,37]. Scientific efforts investigating asthma endotypes, identifying potential biomarkers, and dissecting disease heterogeneity have been enhanced by an undeniable shift towards a holistic systems-based perspective in asthma precision medicine. As a result, apart from various relatively small-scale but highly informative efforts, additional large and centralized consortia conducting comprehensive systems studies, such as the Unbiased Biomarkers for the Prediction of Respiratory Disease Outcome (U-BIOPRED) [38,39] and the Severe Asthma Research Program (SARP) [40,41], have tremendously aided to stratify asthma patients and tailor new targeted treatment options. Although it is far from the focus of this review to cover the extensive relevant literature, some meaningful insights from genomics, epigenomics, transcriptomics, proteomics and metabolomics studies are provided below to better understand the molecular basis of asthma heterogeneity.

### 3.1. Genomics-Epigenomics

Large-scale genome-wide association studies (GWAS) have identified numerous genetic variations and genomic loci possibly associated with asthma characteristics [42,43]. However, only a handful of these results—mainly related to genomic variations identified in the 17q12-21 locus (*ORMDL3* and *GSDMB* genes)—are considered to be reproducible, especially in the case of early-onset childhood asthma [42,44,45]. Additionally, an important number of conducted pharmacogenomic studies have highlighted a potential link between some genetic variations within the *ADRB2* gene and altered drug response against long-acting beta agonists in asthmatic children [46,47,48], a notion that merits further investigation towards a personalized medicine approach in asthma treatment.

Given the intrinsic multifaceted features of asthma, the role of environment-related epigenetic DNA changes associated with asthma manifestation has gathered considerable attention, providing complementary information to genomic research through epigenome-wide association studies (EWAS) and meta-studies of DNA methylation. A meta-analysis of 13 cohorts has identified thousands of differentially methylated sites of cytosine–guanine dinucleotides (CpGs) in the blood of newborns and children, depending on the maternal smoking status [49]. Some of the corresponding genes are asthma-related, indicating a possible exposure–disease relationship in the case of maternal smoking. Two additional, large meta-EWAS focusing on childhood asthma have identified specific differentially methylated CpGs of interest in newborn and child blood samples [50,51]. Xu et al. identified differentially methylated CpG sites in asthmatic children and their corresponding gene expression profiles associated with T-cell cytotoxicity and activation of eosinophils [50]. Moreover, Reese et al., by analyzing blood samples from asthmatic newborns and children, highlighted CpG sites and genomic regions as potential biomarkers of asthma risk and related immune responses. The resulting CpGs of interest were also replicated to some extent in datasets of nasal epithelium cells and eosinophils [51]. Lastly, an interesting DNA methylation study including asthmatic (*n* = 74) and non-asthmatic adults (*n* = 41) has revealed a putative epigenetic regulatory role of 17q12-21 locus associated with asthma risk and *ORMDL3* overexpression in the airways of asthmatic patients [52].

### 3.2. Transcriptomics–Proteomics

Transcriptomic studies have played a keystone role in systems-based investigations of asthma, providing crucial information about gene expression association to observed phenotypes and relevant Th2-high and non-Th2 endotypes [53,54]. Although highly indicative in some cases, transcriptomics cannot fully explain the observed protein expression. Therefore, proteome studies have also been undertaken to complement and enhance research endeavors regarding inflammation and immune response biomarkers in asthma. These specific fields of systems studies in asthma have been pursued by large-scale consortia such as U-BIOPRED and SARP initiatives.

In particular, the U-BIOPRED consortium has provided invaluable information regarding asthma severity in adults and children, both at the clinical and molecular level. Stable patient clusters—characterized by a distinct sputum transcriptomic and proteomic profiling—have been identified using a small subset of clinical-physiologic variables associated with asthma severity [55]. A transcriptomic and gene set enrichment analysis of epithelial brushings and bronchial biopsies from patients with moderate-to-severe asthma revealed distinct subgroups of patients associated with eosinophilic or non-eosinophilic inflammatory phenotypes [56]. At the same time, Kuo et al. explored the differential gene-expression landscape between eosinophilic and non-eosinophilic inflammation in sputum samples and obtained phenotypically relevant clusters based on transcriptomic molecular features [57]. Additionally, gene expression and pathway analysis based on transcriptomic profiling of peripheral blood in the U-BIOPRED cohorts has highlighted gene signatures and implicated biological pathways associated with differential asthma severity but also in comparisons between asthmatics and healthy controls [58]. A cross-sectional observational study of adult asthmatics from U-BIOPRED has identified differentially enriched gene signatures between adult-onset and childhood-onset severe asthma in nasal, sputum and bronchial samples [59]. These signatures reveal differences in eosinophilic inflammation, mast cell presence and lung injury. Moreover, sputum proteomics and airway cell transcriptomics analyses from U-BIOPRED patients have been successfully implemented to explore the distinct molecular features of smoker, ex-smoker and non-smoker severe asthmatics, despite the similar clinical manifestation of asthma in these cases [60]. In 2019, Schofield et al. presented a proteomic-based clustering of asthmatic patients from the U-BIOPRED cohort, providing a valid molecular basis for the distinction of granulocytic inflammation in asthma presentation [61].

The SARP consortium is another pivotal ongoing effort that provided valuable insights into the transcriptomic and proteomic characteristics of asthma manifestation. By performing a gene co-expression network analysis in 155 asthmatics and healthy controls from the SARP cohort, Modena et al. attempted to identify underlying mechanisms in severe asthma [62]. The expression of several hub genes related to epithelial growth and repair, located near the 17q12-21 locus, was notably decreased in severe asthmatics. Additionally, there are remarkable efforts towards a proteomic characterization of asthma-associated inflammation and immune responses inside the SARP consortium. By implementing machine learning models based on cytokine measurements from bronchoalveolar lavage (BAL) samples from severe and non-severe asthmatics, Brasier et al. explored cytokine expression patterns associated with distinct asthma phenotypes based on inflammation characteristics [63]. Furthermore, in two different studies, Hastie et al. shed light on the complex association of inflammatory protein mediators and asthma severity by stratifying patients based on eosinophilic, neutrophilic or other non-eosinophilic characteristics and exploring differential protein expression of cytokines, chemokines and growth factors [64,65].

### 3.3. Metabolomics

Metabolomics have also emerged as a continuously evolving and rapidly ascending field of -omics approaches in asthma research [66]. Blood plasma and serum, urine and exhaled breath condensates (EBCs) pose as informative and easily accessible types of biological samples for analysis by NMR, GC-MS or LC-MS approaches in asthma metabolomics [66]. In particular, the case of “breathomics”—in which EBCs are analyzed by GC-MS, NMR or e-Noses [67] for volatile organic compounds (VOCs) profiling—has gathered a notable amount of attention. In a study involving asthmatics (*n* = 35) and healthy controls (*n* = 23), by implementing logistic regression models in breath sample data analyzed by GC-MS, specific VOCs including various alkanes could classify asthmatics and controls, as well as differentiate eosinophilic and neutrophilic phenotypes [68]. Similarly, in another study, utilizing random forest models and unsupervised k-means clustering in NMR spectra from EBCs has provided a solid model of distinctive features, including formate and hydroxybutyrate, towards the differentiation of asthmatics (*n* = 89) and controls (*n* = 20), along with a clinically relevant formation of patient clusters partially corresponding to previously established neutrophilic endotypes [69]. Compared to GC-MS and NMR that measure individual VOCs, e-Noses can only identify signal patterns of VOC mixtures. Nevertheless, e-Noses have provided noteworthy results in asthma research throughout the years [70,71]. Attempting to evaluate the ability of e-Nose-derived VOC patterns to classify asthmatics and controls, along with predicting patient steroid response, van der Schee et al. studied mild-to-moderate subjects and healthy controls [70]. e-Nose VOCs analysis could classify asthmatics and controls with lower but similar accuracy to FeNO and eosinophil counts in sputum as well as predict steroid response with a crucially greater accuracy than both alternatives. A more recent study, based on e-Nose VOCs from a U-BIOPRED subset of adults (*n* = 78), managed to identify three clusters of patients with distinct inflammatory characteristics (blood neutrophils and eosinophils percentages) and OCS use [71], along with assessing cluster stability over time.

Apart from EBCs, blood plasma and serum from asthmatic patients is another useful biological sample source. A multivariate regression-based analysis of serum NMR spectra from asthmatics (*n* = 39) and healthy controls (*n* = 26) provided a panel of potential asthma biomarkers (increased levels of methionine, glutamine and histidine; decreased levels of formate, methanol, acetate, choline, O-phosphocholine, arginine and glucose compared to healthy controls) and revealed implicated hypermethylation, hypoxia and immune response pathways [72]. Another study by Reinke et al. provided different “metabotypes” of asthma by analyzing through high-resolution LC-MS serum samples from asthmatics (mild, moderate, severe) and non-asthmatic controls [73]. Finally, in a relatively recent analysis of blood plasma from 237 children (46 with current asthma and 191 controls), although a partial least squares discriminant analysis could not identify significant between-group metabolome changes, individual regression for each metabolite yielded some interesting results [74]. A potential association of current childhood asthma with metabolite perturbations related to nicotinamide and pyrimidine metabolism, production of bile salts and heme catabolism was observed. Furthermore, higher levels of p-cresol sulfate—a microbial metabolite from the gut microbiome—were linked to decreased odds of current asthma, and this association was replicated in an independent cohort.

## 4. The Human Microbiome in Asthma Research

In addition to the above-mentioned intensive studies, a substantial body of research aims towards the investigation of the asthmatics microbiome and its relationships to environmental stimuli, disease subtypes and medication, as all these aspects are considered very crucial for further understanding asthma in the context of precision medicine [75,76,77].

### 4.1. The Importance of the Initial Host Microbial Colonization

Several consistent findings have generally acknowledged a crucial role of the microbiome in atopy and asthma [78,79]. Epidemiological research has indicated that a rich microbial environment in early life confers protection against the development of several chronic inflammatory respiratory disorders [80,81]. The well-known “hygiene hypothesis” from 1990, properly adapted to incorporate the relevance of host microorganisms, suggests that perinatal microbial exposure along with early life contact with environmental microbes are necessary to ensure proper colonization of distinct body habitats (primarily gut, respiratory tract, skin, genital tract, etc.). This, in turn, is essential for the development of healthy immune functions, especially of tolerance, as well as for the competitive protection against pathogenic microbes [82,83]. The time and mode of childbirth, maternal age, diet, hospitalization, body mass index (BMI), smoking status, socioeconomic status, breastfeeding and antibiotic use all shape the establishment of the infant microbiome, which reaches its long-term stability for many microbial species at approximately two years of age [84].

In asthma, the positive or negative interplay between the exogenous microbiome and the microbiome of mucosal respiratory tract surfaces has been shown to play a persistent role in influencing the airways’ physiological equilibrium. For example, high microbial diversity in the environment has been associated with lower asthma risk, particularly in children exposed to farming [85,86]. On the other hand, viral respiratory infections with rhinovirus and respiratory syncytial virus during infancy have been shown to be crucial in driving permanent wheeze and bronchiolitis that often precedes full-blown [87]. Furthermore, a recent investigation of the exogenous mycobiome and bacteriome in the indoor dust of severe asthmatic patients identified notable relationships, with more medically relevant microbiome and higher mycobiome diversity to be associated with distinct inflammatory asthma subtypes [88].

### 4.2. The Key Role of the Gut and Airway Microbiota Cross-Talk in Asthma

The gastrointestinal tract is by far the most abundant microbial ecosystem in the human body. In the last decade, there has been an explosion of studies, strongly associating gut microbiota dysbiosis with the pathophysiology and clinical manifestations of a wide range of diseases, from bowel inflammatory disorders, mental diseases and cancer to allergy, asthma and respiratory infections, including coronavirus disease [89,90,91,92,93]. It is well known that gut microbiota acts as a major modulator of immune training and functions through its complex interactions with the gut-associated lymphoid tissue, not only locally but at remote sites as well, including the mucosal surfaces of the respiratory tract [94,95,96]. Furthermore, gut bacteria interactions with therapeutic drugs can modulate their availability, efficacy and host response to treatment [97,98].

A higher abundance of certain bacterial taxa (*Faecalibacterium*, *Lachnospira*, *Rothia*, *Bifidobacterium* and *Akkermansia,* among others) in the gut microbiome, especially during the first month of life, has been shown to associate with protection against allergic sensitization and allergic asthma [99,100,101]. A potential link between the gut microbiota and atopy is its influential role in the induction of Tregs, a subpopulation of T cells that modulate immune system activity, maintain tolerance to self-antigens, and prevent autoimmune disease development [102,103]. Some of the commensal gut bacteria have been also shown capable of modulating the Th type 1/2 (Th1/Th2) balance [104] or directly stimulating Th17 cell differentiation [103]. Furthermore, gut microbiome is an important producer of key metabolites, such as short-chain fatty acids (SCFAs), polyunsaturated fatty acids, tryptophan, gaseous molecules, etc., all actively implicated in host physiology [105]. For example, *Clostridium* spp. are producers of propionic acid (PPA) following the fermentation of complex carbohydrate fibers implicated in the modulation of cell signaling, activation of Tregs, reduction in Th2 inflammation, and neurotransmitter synthesis and release [106,107,108]. Additionally, differences in SCFAs have been observed in infants at the age of three months who later exhibited atopic wheeze by age one [99,100,109].

Although the gut microbiome significantly contributes to the immune regulation and host response to allergens, asthma originates in airways, making the respiratory microbiome more relevant to exert a direct, both acute and long-term, effect on disease pathogenesis progress and control. Modern sequencing technologies have revealed that in healthy individuals, the lung is not a sterile organ, but it rather harbors a low in density, yet dynamic, microbial community, dominated by the bacteria phyla *Bacteroidetes* (mostly *Prevotella* and *Veilonella* spp.), *Actinobacteria* and *Firmicutes*. To date, every study on respiratory microbiota in relation to any lung disease, from asthma and chronic obstructive pulmonary disease (COPD) to cystic fibrosis and acute infections, has demonstrated clear differences in bacterial colonization compared to the healthy state and suggested its direct impact on local inflammatory processes [110,111,112]. Therefore, studying the dynamic changes in the composition of the airway’s microbial communities and their association with host and environmental factors can provide critical novel insights into respiratory disease pathogenesis and may redefine clinical management [113].

**Figure 2:**
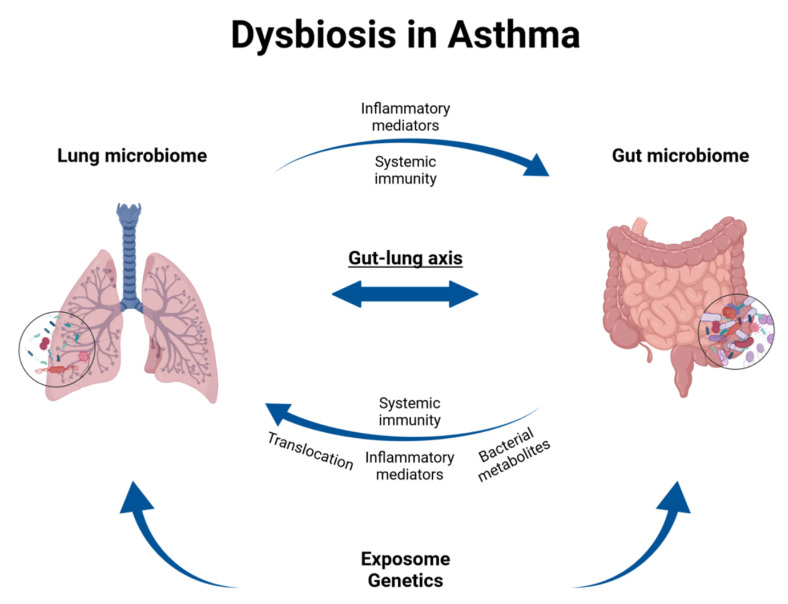
Schematic representation of dysbiosis in asthma. Exposome refers to factors such as environmental microbiota, allergens, air pollution, tobacco smoke, diet, medication and early-life exposures.

There are several lines of evidence supporting the implication of respiratory microbiome in asthma initiation [114,115]. Due to a disruption of the delicate balance between immigration and elimination of bacteria in the lower respiratory tract, asthmatics present altered bacterial composition in their lungs compared to healthy subjects, though the reported alterations are not always consistent in the various studies. Increased *Proteobacteria* populations (especially *Haemophilus*, *Moraxella, Streptococcus* and *Neisseria* taxa) and reduced *Bacteroidetes* and *Fusobacteria* communities within both young and adult patients’ airways microbiome have all been associated with the triggering of inflammatory responses, asthma symptoms, hyper-responsiveness and exacerbation [116,117,118]. Remarkably, the detection of asthma-related bacteria, especially after viral infections, in the first few months of life has been associated with developing allergic asthma by the primary school age [80]. As mentioned above, asthma may also have its roots in dysbiosis occurring in the gut microbiome that can affect the integrity of the airways microbiome. Conversely, lung microbiome aberrations in asthmatics may modulate the immune responses for microbiota residing in the gut. Such mutual interactions mediated by locally resident microbes, circulating active biomolecules (microbial metabolites, pro-inflammatory factors) and mucosa-associated lymphoid tissue in concurrence with systemic immunity has led to the concept of the “gut–lung axis” (Figure 2), pointing out that microbiomes at both niches may be significant contributors to the pathogenesis of asthma [90,119]. However, further studies are needed to fully understand the mechanism of such cross-talk [120].

While most microbiome studies in asthma have focused on the identification of bacterial communities, recent advances in sequencing approaches and taxonomic databases have allowed the broad characterization of all the microorganisms within both gut and airways ecosystems. Overall, current knowledge supports that the host microbiome implication in asthma pathogenesis and heterogeneity is not limited solely to bacteria but also involves various other microbes, such as fungi and viruses, that altogether interact and shape the dominant host microbiota communities which either maintain the appropriate healthy balance or lead to dysbiosis and consequently to asthma manifestation and exacerbations. For instance, several lines of evidence have suggested a possible contribution of fungi communities in immunomodulatory mechanisms developed in early life [121], while differences between the airways fungi composition in asthmatic patients and healthy controls have also been widely reported [122].

### 4.3. Microbiome and Crucial Asthma Risk Factors

In addition to the well-studied role of environmental microbes, extensive research has also underlined the importance of other host exposome features, i.e., factors a person is exposed to throughout his life (including drugs, tobacco smoking, pollutants, allergens, diet, etc.) in asthma [123]. Although there is still no clear mechanistic evidence on how exposome affects asthma, many studies have recently indicated either direct or indirect contribution to airway microbiome restructuring by either harmful or beneficial stimuli with subsequent consequences in lung functionality and host immune training and modulation [77]. As the detailed discussion of these aspects is considered to be beyond the scope of the present review, we only briefly focus below on two well-established asthma risk factors, namely tobacco smoking and obesity and their association with airway microbiome alterations.

#### 4.3.1. Tobacco Smoking

Tobacco smoking has been extensively studied as it is recognized to induce non-Th2 neutrophilic inflammation, leading to severe steroid-resistant symptoms and mortality in asthma patients [60,124,125]. Smoking cessation programs are considered important therapeutic interventions towards improving the clinical outcomes in asthmatic smokers [126]. Regarding microbiome, tobacco smoking per se has been associated with alterations of both gut and upper respiratory tract microbial communities in healthy individuals [127,128,129,130,131,132]. More specifically, in a large meta-analysis, the oral microbiome composition of current smokers compared to those who never smoked or former smokers has proved to be substantially differentiated [128]. Biedermann et al. showed that healthy individuals undergoing smoking cessation were characterized by an increase in bacteria in the gut [133]. However, the relationship between smoking and bacterial profiles in the respiratory tract of patients suffering from asthma is still not well studied. Çolak et al. showed that smoking-induced alterations in the lower airway microbiome might be related to an increased risk and severity of pulmonary disorders in asthmatic smokers [134]. Munck et al. identified differences in bacterial diversity between tobacco smokers with asthma compared to healthy non-smoking controls, but they failed to demonstrate any association between smoking cessation and microbial alterations, probably due to the small sample size, short period of follow-up and distinct asthma subtypes [135].

#### 4.3.2. Diet and Obesity

Long-term dietary patterns with energy-dense nutrient-poor foods and primary obesity are also associated with an increased incidence of asthma in adults [136,137]. Obese asthma has been repeatedly included in asthmatic clusters mostly characterized by female, adult-onset, neutrophil-mediated and severe asthma, although there are also fewer cases of obese asthmatic patients clustered in other asthma subgroups such as atopic asthma [137,138,139]. Although the exact mechanisms that link asthma with diet and obesity have not been clarified so far, interventions on obese asthmatics, including diet modifications or bariatric surgery, have led to efficient asthma control [139,140]. Increasing evidence indicates that the gut microbiome may play a significant role in obese asthma [139]. Obesity and diet can alter the gut microbiome composition [141,142,143], whereas other pathological conditions directly linked with obesity, such as hyperglycemia, insulin resistance and systemic inflammation, have a bidirectional relationship with the gut microbiome [144]. In turn, the imposed gut microbiome alterations could induce asthma via the gut–lung axis and through the production of bacterial-derived or modified metabolites, such as SCFAs. More clear indications of the microbiome involvement in obese asthma phenotype include findings from studies in severe asthmatics, where BMI is associated with different microbiome compositions, especially with *Bacteroidetes* (including *Prevotella* species) and *Firmicutes* (e.g., *Clostridium* species) [137,145]. Most importantly, in severe asthmatics, the lung and the gut microbiome present alterations between obese and non-obese subjects [146]. In the study by Michalovich et al., an additive effect of asthma and obesity has been shown to formulate the gut and lung microbiota inhabiting obese asthmatics. Interestingly, in the same study, *Akkermansia muciniphila*, which has been shown to present protective effects in obese models, exhibits reduced levels in the gut microbiome of obese compared to non-obese asthmatics [147].

## 5. Airway Microbiome Correlations with Asthma Subtypes

Several studies (Table 1) providing detailed clinical features of asthmatic participants have shown distinct associations of airway microbial variation with different asthma phenotypes and endotypes [117,118,146,148,149,150,151,152,153,154,155,156,157]. Most of these works compare the microbial structure amongst groups of patients with distinct severity, clinical features or inflammatory background versus healthy controls using specific metrics mainly represented by alpha diversity (a measure of species richness and evenness) and beta diversity (a measure of dissimilarity between different communities/samples). Typical variables that are examined include the total bacterial abundance (bacterial burden in samples), the microbial richness (number of bacterial species in a community), the microbial evenness (level at which the species within a studied community are evenly distributed) as well as the relative abundance and the predominance of particular bacteria at family, genus or species levels.

An early study examining the microbiome within the sputum samples of 28 severe asthma patients has related the predominance of *Moraxella catarrhalis* or of species belonging to *Haemophilus* and *Streptococcus* genera with neutrophilic airway inflammation [117]. Huang et al., evaluating the bronchial airway microbiome of 40 severe asthmatic patients, concluded that there was negative correlation between the bronchial eosinophil numbers and the relative abundance of certain bacteria belonging to the *Proteobacteria* phyla (*Moraxellaceae* and *Helicobacteraceae* family members), as well as to a positive correlation between the bronchial eosinophil numbers and proportions of *Streptomyces* and *Propionicimonas* species [146]. Zhang et al., through the comparison of the lower airway microbiome of severe and non-severe asthmatics with that of healthy controls, found that *Firmicutes* were increased in severe asthmatics compared to controls, and more specifically, *Streptococcus* spp. were associated with recent-onset asthma and sputum eosinophilia [149].

Sverrild et al. examined the BAL microbiome profile of a cohort of 23 asthmatic patients. The main finding from this study was that the relative abundance of specific bacterial genera (*Aeribacillus*, *Halomonas*, *Neisseria*, *Nesterenkonia*, *Rothia*, *Shewanella*, *Sphingomonas*, *Actinomyces*, *Bacteroides* and *Virgibacillus*) differed significantly between eosinophilic asthmatics and healthy controls. Significant differences were also shown regarding the relative abundance of specific bacteria (*Flavobacterium*, *Phenylobacterium*, *Brevundimonas*, *Bradyrhizobium*, *Sediminibacterium*, *Gemella*) between neutrophilic asthmatics and healthy controls [150]. Another study that included patients suffering from severe (*n* = 25) and non-severe asthma (*n* = 24) resulted in enriched *Actinomycetaceae* and *Enterobacteriaceae* family members in eosinophilic as opposed to non-eosinophilic asthma patients [151].

Two independent studies comparing the airway microbiome of neutrophilic with non-neutrophilic asthmatic patients revealed a significantly lower proportion of *Actinobacteria* and *Firmicutes* and a significantly higher proportion of *Haemophilus influenzae* in neutrophilic asthmatics [148,152]. Interestingly, in the study by Taylor et al., among 167 asthma patients with neutrophilic, eosinophilic, paucigranulocytic or mixed granulocytic inflammatory endotypes, application of principal coordinates analysis (PCoA) resulted in distinguishing neutrophilic samples from the rest endotypes based on their microbiome composition. The greatest differences in composition were observed between neutrophilic and eosinophilic asthma patients. The neutrophilic patients also displayed the smaller diversity, richness and evenness in their sputum microbial composition. Significant differences were once more observed in the airway bacterial taxa between the different endotypes, with neutrophilic asthma exhibiting enrichment in pathogenic taxa. Namely, high abundance of *Haemophilus* and *Moraxella* was observed in neutrophilic patients, whereas negative correlation was observed between the eosinophilic percentage and *Haemophilus* genera. The abundance of *Gemella*, *Rothia* and *Porphyromonas* in neutrophilics decreased compared to the other examined inflammatory endotypes, while a significant correlation was observed between *Streptococcus I*, *Neisseria* and *Gemella* genera and eosinophilia [153].

Ghebre et al. investigated the microbiome profiles of patients suffering from asthma during exacerbations, resulting in biological clusters, each one including patients with distinct bacterial composition associated with different inflammatory endotypes. Asthma patients experiencing exacerbations with increased blood and sputum neutrophils were grouped in the same cluster characterized by considerable proportions of the bacterial phylum *Proteobacteria*. In contrast, asthma patients with increased eosinophils in blood and sputum were clustered together with a higher proportion of *Bacteroidetes* [154].

Accordingly, a study conducted in Northeast China including patients with mild-to-moderate asthma showed a significant decrease in microbial diversity, richness and evenness in the sputum of non-eosinophilic compared to eosinophilic asthmatics. Moreover, a distinct microbial taxonomic profile characterized the two groups of patients. More specifically, *Glaciecola* and *Helicobacter* were more abundant at the genera level, whereas *Deinococcus*, *Scardovia*, *Bifidobacterium* and *Desulfobulbus* were less abundant in eosinophilic compared to non-eosinophilic asthmatics [155].

Two other works examining the composition of the bronchial and sputum microbiome of asthmatic patients revealed lower bronchial and sputum bacterial burden in patients with Th2-high asthma compared to non-Th2 asthma patients [118,156]. Finally, in a recent longitudinal study, Abdel-Aziz et al., by examining the sputum microbiome profile of patients characterized by severe asthma phenotype, concluded into two distinct microbiome-driven clusters which, among others, are also characterized by different neutrophilic content. The cluster characterized by higher sputum neutrophilic percentage and greater asthma severity generally presented lower microbial richness and diversity as well as a trend toward an increased relative abundance of some pathogenic species (such as *Haemophilus influenzae*, *Moraxella catarrhalis* and *Streptococcus pseudopneumoniae*) and decreased abundance of species related to genera *Veillonella*, *Prevotella*, *Rothia*, *Haemophilus* and *Neisseria* as compared to the cluster characterized by low-neutrophilic content [157].

As a general remark, neutrophilic asthmatics demonstrate a lower airway microbiome diversity compared to healthy controls and other disease endotypes. Actually, a crosstalk between neutrophil regulation and microbiota structure has been shown either in health or disease [158]. For example, it has been shown that microbial metabolites may either enhance or suppress neutrophilic functionality, and this interaction may be involved in the progression of chronic inflammation-related diseases [159]. The involvement of microbial dysbiosis in patients with severe non-Th2 asthma is also supported by studies showing that treatment with antibiotics, including macrolides, such as azithromycin, may result in improved disease control, airway hyper-responsiveness and inflammation, especially in neutrophilic asthmatics [151,160]. The microbiome profile in patients with the other non-Th2 inflammatory endotypes has not been widely studied so far, but it is probably distinct from the neutrophilic asthma-related microbiome.

In addition to the above investigations concentrating mainly on the analysis of airway bacterial communities, further efforts aimed to examine the poorly considered associations between airway mycobiome and asthma. Sharma et al. reported a combination of identified fungi biomarkers along with other clinical features for distinguishing asthma endotypes. More specifically, a lower fungal diversity in asthma patients with Th2-high compared to non-Th2 inflammation was found in samples of bronchial brushes. Enrichment of *Trichoderma* species was found in Th2-high asthmatics, while an association between *Alternaria*, *Aspergillus* and *Fusarium* species and neutrophils was observed. At the same time, enrichment of fungal genera (*Aspergillus*, *Cladosporium*, *Fusarium*, *Penicillium*, *Trichoderma* and *Mycosphaerella*) in BAL of asthmatics with T2-high inflammation was identified [161]. Recently, Huang et al. also attempted to characterize the airway microbiome of untreated and ICS treated patients focusing on both mycobiome and bacteriome. The main findings from this study include distinct mycobiome composition and biodiversity after comparing the two groups of asthmatic patients and healthy controls; furthermore, network analysis indicated unbalanced associations between bacteriome and mycobiome, suggesting asthma-specific inter-kingdom alterations [162].

In general, there is a distinct microbiome profile corresponding to the different asthma inflammatory pathways, and certain bacteria taxa could be considered as candidate markers for asthma endotypes. The presence of pathogenic bacterial species belonging to *Proteobacteria* phyla or *Gammaproteobacteria* class, including species from *Haemophilus* and *Moraxella* genera, have been shown to be more dominant in the airway microbiome of patients with neutrophilic inflammation. On the other hand, studies of asthmatics presenting Th2-high inflammation, particularly eosinophilic phenotype, demonstrated more heterogeneous results concerning their microbiota content, although a correlation of bacteria belonging to *Actinobacteria* phylum with eosinophilic asthma has been observed. This lack of clear associations between specific bacteria and Th2-high inflammatory endotypes could be attributed to a larger contribution of exogenous microorganisms or other exposome factors such as allergens, rather than the host bacteriome, in the perpetuation of Th2-high inflammation.

## 6. Relationships between Asthmatics Airway Microbiome and Treatment

The results accumulated thus far raise expectations concerning the exploitation of microbiome characterization in the selection of a precision strategy for asthma management. This approach must take into consideration the potential interactions between the patient’s microbiome and the administered medication [97,98].

In this direction, several studies have tried to clarify the effect of therapeutic drugs on airway microbiome structure and vice versa. A study by Denner et al. has shown that the increasing administration of either ICS or a combination of OCS and ICS is associated with alterations of the bacterial microbiome in epithelial brushes and specifically causes an increase in *Proteobacteria* and a decrease in *Bacteroidetes* and *Fusobacteria* at the phylum level. In addition, a decreased abundance of *Veillonella* species was related to ICS, whereas an increase in *Pseudomonas* species was associated with OCS administration [163]. Accordingly, Taylor et al. reported a significant correlation of bacterial diversity in induced sputum of patients with moderate-to-severe asthma and ICS dose [153]. Furthermore, Sharma et al. found a differential abundance of fungi belonging to genus *Penicillium* in BAL and bronchial brushings between ICS treated and non-treated asthmatics [161]. The crucial role of medication in the structure of asthmatics’ microbiome was underlined by studies that did not detect any differences, at the phyla level, between healthy controls and steroid-naïve patients [164]. However, McCauley et al. showed that nasal *Moraxella* was associated with increased exacerbations and eosinophil activity in asthmatic children, but, although treatment with omalizumab resulted in reduced exacerbations, the pathogenic nasal airway microbiota was not significantly modified after treatment [165]. In addition, Martin et al. could not identify significant differences in sputum bacterial load or overall community composition between low- and high-dose ICS treatment of asthmatic patients. However, they observed an association between high-dose fluticasone propionate and increased abundance of the pathogen *Haemophilus parainfluenzae* [166].

On the other hand, to investigate the contribution of airway microbiota in the observed heterogeneity of asthmatic patients’ responsiveness to treatment, several studies compared the composition of microbial communities between responders and non-responders. Goleva et al. found that the bacterial content in BAL of asthmatic patients that were sensitive or resistant to corticosteroids differed significantly. The majority of non-responders presented increased proportions of microorganisms belonging to phyla *Actinobacteria* and *Proteobacteria* and significantly lower proportions of bacteria belonging to *Fusobacteria* phylum and *Prevotella* and *Veillonella* genera as compared to healthy controls. Most responders presented increased proportions of bacteria from phylum *Proteobacteria* and significantly reduced proportions of bacteria belonging to genera *Prevotella* and *Veillonella* compared to healthy controls. Additionally, bacteria belonging to *Neisseria*, *Haemophilus*, *Simonsiella*, *Campylobacter*, *Leptotrichia*, *Tropheryma*, *Leuconostoc* and *Megasphaera* genera were identified in a subset of non-responders but were not present in corticosteroid-responsive asthmatics. On the other hand, many bacteria belonging to genera *Bradyrhizobium*, *Aquabacterium*, *Limnobacter*, *Pasteurella*, *Fusobacterium* and *Streptophyta* were only identified in a subset of responders but not in non-responders [167]. These results corroborated previous evidence supporting a positive correlation between *FKBP5* gene expression—a steroid response biomarker—and lung microbiome composition [146]. Durack et al. showed that in initially ICS-naïve asthmatics, ICS-responsiveness is associated with distinct features of the bronchial bacterial microbiota before treatment, with the responders’ microbiome being more similar to that of healthy controls. Non-responders presented higher abundance of *Microbacteriaceae* and *Pasteurellaceae*, whereas *Streptococcaceae*, *Fusobacteriaceae* and *Sphingomonodaceae* were enriched in responders [118]. A later study examining the sputum microbiota of asthmatics before and after ICS treatment revealed that the composition of sputum microbiota showed greater deviation in ICS non-responders as compared to ICS responders [156]. Finally, Thorsen et al. found that in preschool children presenting asthma-like symptomatology, the airway microbiota was a modifying factor concerning the efficacy of azithromycin treatment during recurrent episodes [168].

Overall, many of the aforementioned studies indicate that the microbiome composition may trigger corticosteroid resistance or influence the efficacy of corticosteroid treatment. Among the presented results, we could distinguish findings indicating a higher relative abundance of bacteria belonging to the phylum of *Fusobacteria* in corticosteroid responders and lower proportions of the same bacteria phylum in non-responders. However, further research should be conducted in the specific field to conclude to useful and valid microbial-markers that could be exploited in the future as prognostic signatures for resistance or response to asthma treatments.

## 7. Integration of Microbiome and Multi-Omics Data from Asthma Patients

Although several studies have thus far investigated the role of host microbiome in asthma and revealed a significant number of microbial biomarkers towards the elucidation of disease heterogeneity, the field remains still in a relatively infant stage. Large-scale, longitudinal studies involving high-quality metagenomic and other host -omics data from asthmatic patients and carefully selected control groups are essential to further enrich current knowledge. In particular, multi-level integrative explorations should be intensively pursued, as understanding the complex interactions and networks of multi-layered molecular data offers a more complete insight into the whole picture than the mere sum of the pieces involved. To that end, during the last decade, there have been notable—yet scarce—efforts towards a more comprehensive, integrative, multi-omics investigation involving microbial abundances as a core focus element. Additionally, a rising number of large-scale initiatives involving microbiome data have been unfolding during the last years, aiming towards a systems-based holistic investigation of asthma classification and severity.

### 7.1. Microbiome and Host Gene Expression Data Integration

Two of the earliest attempts involved a relatively small subset of asthmatic versus healthy (eight and six, respectively) children and adolescents from the AsthMaP cohort [169] and could be characterized as complementary to each other [170,171]. Both studies implemented a meta-transcriptomic-host transcriptomic gene expression analysis performed on shotgun RNA sequencing data from nasal brushings, followed by a rudimentary data integration process. In both cases, microbiome-host read separation was conducted in silico through appropriate bioinformatic approaches. The first study by Castro-Nallar et al. focused mainly on the microbial composition of patients and controls, defined as the combined effect of microbiota and their corresponding gene expression. Asthmatic patients presented a less diverse microbial profile compared to controls, with *Moraxella catarrhalis* being the prevailing species in most asthmatic samples. Subsequent PCoA revealed that patient samples highly abundant in this specific species tend to cluster together and differentiate from controls and asthmatic patients with low levels of *Moraxella catarrhalis.* Given the prevalence of *Moraxella catarrhalis* in most asthmatic patients, the host gene expression for a particular gene signature known to be associated with immune response against this species was evaluated and found to be able to distinguish asthmatic and control samples. The strength of host immune response, as determined by the expression profile of this particular gene signature, was mostly concordant with *Moraxella catarrhalis* abundance. In the complementary study of Pérez-Losada et al., a similar dual transcriptomic profiling was implemented, this time focusing mainly on the functional characteristics of the airway microbiome and their association to host immune and inflammatory response. Taxonomic profiles previously identified by Castro-Nallar et al. were also utilized. A differential host gene expression between asthmatics and controls was retrieved, and this core asthma signature was associated with upstream regulatory mediators of inflammatory and immune responses. Additionally, the study highlighted differences in microbial metabolic functions between groups, mostly connected to basic metabolism, nitrogen metabolism, central carbohydrate metabolism, sugar alcohols, xenobiotic biodegradation, glycan biosynthesis and microbial adhesion. Finally, a multivariate approach was implemented to discover associations between microbial metabolic functions, taxa abundance and the identified host regulatory mediators in asthmatics. As a result, a significant positive association of host *IL1A* upregulation and increased microbial adhesion in asthmatics was documented, along with a potential link between *Proteobacteria* abundance and *IL1A* expression changes.

Another study published in the same year focused mainly on the bronchial microbiome of patients with severe corticosteroid refractory asthma (*n* = 40) and its association to disease severity and inflammatory characteristics [146]. Healthy controls (*n* = 7) and patients with mild-to-moderate asthma (*n* = 41) were also included. Microbial composition and function prediction based on 16S rRNA gene arrays and appropriate data analyses were conducted parallel to host gene expression investigation from separate epithelial brushes. Three gene expression-based signature scores regarding a) steroid response (*FKBP5* gene), b) Th2-high inflammation and c) Th17 inflammation were used to explore a possible taxa abundance interaction with host gene expression. *Actinobacteria* abundance and increased bacterial diversity were positively correlated with *FKBP5* expression and, therefore, possibly steroid response. On the other hand, although no significant associations were detected between taxa and the studied Th2-high inflammation signature, certain *Proteobacteria* (*Pasteurellaceae*, *Enterobacteriaceae*) and *Firmicutes* (*Bacillaceae*) abundances were positively connected to Th17 inflammation, potentially involved in neutrophil recruitment and airway inflammation.

Recently, an integrative network-based study explored the interplay between upper and lower airway microbiome and host transcriptome in cases of severe persistent childhood asthma (*n* = 27) and carefully selected healthy controls of similar age (*n* = 27) [172]. Nasal and bronchial samples were collected from patients for 16S rRNA gene sequencing and RNA sequencing analyses, while nasal samples were obtained from controls due to restrictions related to the invasive nature of bronchoscopy. Differential gene expression between nasal and bronchial samples of patients led to the identification of differentially enriched gene ontology functional terms regarding cilium assembly, morphogenesis and movement, along with inflammatory response. Additionally, many genera were differentially abundant between patient nasal (*Corynebacterium*, *Staphylococcus* and *Moraxella*) and bronchial (*Veillonella*, *Prevotella*, *Streptococcus* and *Neisseria*) samples. By implementing a custom method based on permutation–renormalization and bootstrapping of two correlation measures and two dissimilarity measures, the authors constructed nasal microbiome, bronchial microbiome and nasal–bronchial microbiome networks in order to explore underlying microbiome interactions. Hub genera, such as *Moraxella*, *Alloiococcus* and *Corynebacterium,* were detected in patient nasal samples, while no hub members were identified in bronchial samples. Examining the relations between nasal and bronchial patient microbiota revealed that *Porphyromonas* in bronchial samples and nasal *Corynebacterium* had the highest number of associations with nasal and bronchial genera, respectively. Two additional microbiome–transcriptome networks in the nasal and bronchial patient samples were created using a highly similar computational method. As a result, *Actinomyces* was found to be negatively associated with the expression of 157 bronchial genes involved in inflammatory response, possibly suggesting a protective role of this hub genus against bronchial inflammation. On the other hand, nasal *Corynebacterium* interacted with an important number of genes; however, no terms enriched in those genes could be identified as significant. Next, differentially expressed genes and abundant microbes were identified between nasal samples from cases and controls. Genes associated with ciliary function and innate immune response were highlighted, along with an increased relative abundance of *Streptococcus* in asthmatic patients. Finally, in contrast to patient samples, *Corynebacterium*, found to be once again a hub core member in the corresponding microbiome–transcriptome network of healthy controls, demonstrated mostly negative interactions with genes significantly connected to inflammatory and immune response terms, along with chemotaxis. This suggests a potential protective mechanism of *Corynebacterium* abundance—host gene expression in healthy controls, possibly impaired in asthmatic children with persistent disease.

### 7.2. Microbiome and Host Metabolome/Proteome Data Integration

Apart from the aforementioned research endeavors, additional studies have attempted integrating microbiome abundances with other levels of host -omics information such as metabolomics and proteomics. A study focused on mite-sensitized allergic asthma in children, through the scope of metagenomics and metabolomics, has explored connections between respiratory microbes and circulating metabolites [173]. A cohort of 32 asthmatic cases and 37 healthy controls provided oropharyngeal swabs for shotgun metagenome sequencing and blood serum samples for NMR spectroscopy to obtain metabolomic profiles. Metagenomic analysis highlighted a more heterogeneous microbial community in cases compared to controls. *Neisseria elongata* was prominently more abundant in the asthma group, while healthy controls were more enriched for *Eubacterium sulci*, *Leptotrichia wadei* and several *Pretovella* spp. Similarly, the metabolic profiling revealed a proline-glutamate chimera and serine enrichment in asthmatics compared to controls, which in turn presented an increase in circulating dimethylamine and dimethylglycine. Additionally, the abundance *of Prevotella sp. oral taxon 306* was positively correlated with levels of dimethylglycine, and a more extensive set of species was positively associated with essential amino acids and energy-related carboxylic acids while negatively correlated with circulating glucose and pyridoxine. The subsequent functional analysis revealed that microbial genes involved in polycyclic aromatic hydrocarbon degradation were significantly enriched in healthy controls. In contrast, bacterial genes related to glycosaminoglycan degradation, histidine metabolism and membrane trafficking were enriched in the asthma group. Of notable importance is the effort of this study to classify studied asthmatics and non-asthmatics based on the available -omics data. Random forest predictive models were implemented separately on metagenomic data, metabolomic data or a combination of both. The combinatorial nature of dual-omics features presented the best results based on total area under the receiver operating characteristic curve (AUC) measurements, and *Eubacterium sulci* emerged as a top discriminatory species between asthmatic and non-asthmatic children.

In addition, a noteworthy multi-omics approach involving metagenomics, metabolomics and proteomics data from the BIOASMA cohort (*n* = 46) has provided important insights regarding obesity-related characteristics in asthmatic children with persistent or episodic asthma [174]. Patients were stratified based on the frequency and severity of the disease as groups of occasional, frequent or persistent asthmatics. Furthermore, a similar stratification led to the formation of three groups of normal-weight, overweight and obese individuals. In addition, 104 non-asthmatic children were included in the study as healthy controls. Inflammatory protein biomarkers were determined in the cohort plasma, along with metabolomic profiles by NMR spectroscopy. SCFAs were specifically quantified by GC-MS. Fecal microbial composition was analyzed by 16S rRNA gene sequencing. Data analysis regarding both obesity and severity included the construction of Partial Least Squares-Discriminant Analysis (PLS-DA) and sparse PLS-DA (sPLS-DA) models. In terms of obesity-associated characteristics, obese/overweight asthmatic children were characterized by lower proportions of plasma acetate, higher levels of leptin and significant presence of a bacteria family from the *Clostridiales* order, compared to normal-weight patients. Regarding asthma severity, children with more persistent phenotypes showed elevated fecal D lactate and D/L lactate ratio, higher proportion of plasma creatinine and presence of an unclassified family of the *Mollicutes* class. At the same time, lower levels of plasma citrate and dimethylsulfone were identified in persistent cases compared to less severe manifestations.

### 7.3. Large Consortia Integrative Multi-Omics Studies

Currently, new large-scale consortia-based multi-omics studies are emerging, potentially aiming to investigate the microbiome involvement in asthma endotyping and/or phenotyping, as well as attempting to integrate metagenomic and other clinical or multi-layer molecular features during asthma progression.

The Genomics and Metagenomics of Asthma Severity (GEMAS) initiative, organized in Spain, aims to assess the role of genomic and metagenomic alterations in asthma exacerbations [175]. A number of 257 children and adults, divided into 137 cases and 120 controls indicated by the presence or absence of severe asthma exacerbations during the last year, are so far enrolled, and the recruitment is still in action. Saliva, nasal and pharyngeal samples have been collected from cases and controls as well as blood samples from a subset of patients. A plethora of clinical characteristics and patient information have been additionally documented. Microbiome diversity will be assessed through 16S rRNA gene sequencing, while genomic features such as Single Nucleotide Polymorphisms (SNPs) will be explored through a next-generation genotyping array. A preliminary comparison of demographic and clinical features between cases and controls has provided an early insight into the expected phenotypes in those groups, indicating the presence of both early-onset allergic Th2-high asthma and adult-onset non-Th2 manifestations. GEMAS analyses and derived results are highly anticipated as, up to now, no clear associations between genomic loci and microbiota presence have been identified in asthma research, despite certain efforts to investigate host immune gene variations and upper airway microbiome changes [77,176]. Apart from the GEMAS initiative, a similar pan-European multi-omics research endeavor named Systems Pharmacology Approach to Uncontrolled Pediatric Asthma (SysPharmPediA) is currently coming to fruition [177]. This study aims to implement an integrative systems medicine approach to characterize the molecular background and possible treatable targets of moderate-to-severe uncontrolled asthma in children and adolescents, on a case-control basis depending on asthma exacerbations (as indicated by OCS use or hospitalization in the past year and calculated asthma control score). A wide range of clinical and demographic data has been collected, along with an impressive number of samples from different sources (feces, saliva, blood/serum, nasal swabs and exhaled breath) in order to accommodate the subsequent -omics analyses. Genomic, epigenomic, transcriptomic, proteomic, metabolomic and metagenomic data will be available from as many as possible samples in each layer, comprising a comprehensive core of single-layer data ready to be computationally integrated. The holistic systems biology approach implemented in this cohort could provide unprecedented knowledge towards a personalized medicine-guided perception of uncontrolled pediatric asthma.

A representative example of a deeper, more comprehensive dive inside the complex multifactorial mechanisms of asthma characteristics in the context of such an extensive initiative was recently presented [178]. Analysis of 120 sputum samples from severe smoking and non-smoking asthmatics, mild-to-moderate asthmatics and healthy controls from the U-BIOPRED adult cohort was conducted to access potential links between endopeptidase expression, microbial composition and inflammatory features, aiming towards a stratification of asthmatics according to risk of SARS-CoV-2 infection. Patients were divided based on a computed endopeptidase expression (TMPRSS2, furin) enrichment score (ES) in high and low ES groups. Between-group comparison of inflammatory markers such as neutrophil/eosinophil count and inflammation proteins, microbial diversity and gene expression of certain exopeptidases (ACE2 and DPP4) and sialyltransferases (ST3GAL4 and ST6GAL1) was performed. An overall higher ES characterized severe asthmatics compared to mild-to-moderate cases and controls, and that ES was significantly correlated with neutrophilia but negatively associated with bacterial α-diversity measures. The high ES group presented with an increased presence of sputum neutrophils but reduced bacterial diversity compared to the low ES group. Moreover, pathogens such as *Moraxella catarrhalis* and *Haemophilus influenzae* were more abundant in the high ES group, along with a higher expression of exopeptidases and sialyltransferases. Finally, an important number of differentially abundant proteins between the two groups was identified—including prominent inflammatory markers (IL-6, MIF, TIMP2, LIGHT, TSG-6 and IL-8)—and subsequent enrichment analysis revealed activated pathways of innate immunity, neutrophil degranulation, cytokine signaling, Toll-like receptor signaling and platelet activation.

Recently, Tyler et al. introduced Merged Affinity Network Association Clustering (MANAclust), an automated pipeline for unsupervised clustering of multi-omics and clinical data, aiming to identify disease endotypes through consensus clustering and feature selection of categorical/numerical variables [179]. To that end, MANAclust was implemented on clinical, microbiome, transcriptomic and methylation data derived from patients included in the Airway in Asthma (ARIA) cohort [180]. A total number of 316 asthmatics and non-asthmatics were included in the analysis. Patients were evaluated regarding the severity of the disease and a multitude of other clinical characteristics. Notably, MANAclust was able to distinguish healthy controls in different consensus groups, mainly corresponding to low prevalence of asthma and high asthma control test scores. Additionally, several endotypes of asthma were identified, including some associated with allergens or upper airway infections. Moreover, a subset of asthmatics presented features of a *Moraxella*-dominated microbiome, accompanied by overall very low a-diversity measurements and methylome–transcriptome profiles associated with leukocyte activation and the immune system. Finally, two subsets of asthma patients presented molecular characteristics statistically independent from the clinical manifestation of the disease, a notion that poses interesting questions in the context of personalized medicine. Table 2 summarizes all microbiome–host multi-omics studies presented above.

## 8. Challenges

Despite the significant progress in asthma research, many challenges remain for the advancement of precision medicine, mainly related to difficulties concerning the unbiased diagnosis, as well as the identification of specific therapeutic targets, particularly for patients with severe non-Th2 inflammation. The exploitation of microbial signatures as biomarkers for asthma classification and potential therapeutic intervention constitutes a promising yet still not clinically applicable approach. Major limitations arise from the variation of the results in different microbiome studies, which is caused by a number of factors [181]. Dissimilarity in patients’ recruitment in terms of age, dietary and smoking habits, comorbidities, environmental exposures or medication may introduce inconsistencies in microbiome composition. Furthermore, the microbiota can change dynamically in the same person over time, even within a day or between different seasons, further perplexing data interpretation. Variations in the followed methodological pipelines (from sampling to DNA extraction, sequencing and data processing) can also introduce potential discrepancies. For example, the sampling source is a very influencing factor, as the different airway niches are inhabited by distinct in density and structure microbial communities. The applied sampling procedure is also extremely crucial, especially in the case of the low mass lung microbiome because of the high cross-contamination risk from the upper airways or external sources. Furthermore, invasive sampling adds extra concern in the investigation of lung microbiome. A cautious study design, well-characterized cohorts of patients, longitudinal follow-up and standardization of procedures are all indispensable for ensuring the quality, reproducibility and clinical relevance of the results.

At the level of high throughput systems biology approaches, it remains challenging to process, analyze, integrate and finally interpret the large volumes of acquired biological data. Even at the core base of single-level microbiome data processing and bioinformatic exploration, robust standardization and best-practice protocols are assertively needed—but not yet fully achieved—in every stage of the analysis [182]. Benchmarking bioinformatics pipelines for 16S rRNA gene sequencing and shotgun metagenomics data analysis can offer useful information on existing computational methodologies’ limitations, advantages, performance and overlap [183,184]. Especially in the case of the low-density lung microbiome analysis, which is inherently more prone to errors, biases and uncertainties [181], gold standard bioinformatic methodologies tackling those issues are required as the field matures.

Moving forward from single- to multi-omics studies, emerging challenges underlying microbiota and host data integration pose as major bottlenecks for a more comprehensive understanding of asthma, although significant progress on analytical, mathematical and computational issues behind integrative approaches has been thus far achieved [185,186,187,188]. A variety of different methods have been proposed based on the available -omics data, sample size and the actual scope of the study [186]. Supervised, semi-supervised or unsupervised computational methodologies can be used depending on whether prior knowledge and annotation of samples are taken or not into consideration [188]. Additionally, integration methods have been categorized as early, intermediate or late based on the stage and manner of integration of the single-omics datasets. Concerning the mathematical analysis, an impressive variety of correlation, multivariate, dimension reduction, clustering, Bayesian, regression-based, network-based and machine learning methodologies have been proposed to enhance integration, assist subsequent interpretation and ultimately overcome restrictions dictated by the data on hand [179,187,188,189,190]. Still, standardization of these methodologies remains extremely difficult due to the wide range of potentially available host -omics data and the inherent microbiome features. For instance, although microbiome data are characterized by specific “statistical” properties, namely compositionality, heterogeneity and sparsity, which should be taken into consideration [191], most of the integrative approaches use conventional statistical methods that may fail to fully address the unique needs and purpose of this type of analysis [192]. A recent effort presenting a well-documented step-by-step protocol of a computational framework towards the integration and interpretation of metagenomics, serum metabolomics and host physiology data [193] could serve as an excellent highly informational paradigm for future multi-omics integration endeavors.

## 9. Future Directions

Microbiome and host multi-omics integration in asthma remains in an early research stage, and therefore, novel perspectives could still be pursued and incorporated to further enhance relative analyses. Gaining information about microbial communities from different perspectives through “meta-omics” methods such as metagenomics, metatranscriptomics, metaproteomics and microbiome metabolomics emerges as an essential step required for a complete taxonomical, functional and even metabolic profiling of the host microbiome for various diseases, hopefully including asthma [194,195]. For example, implementing network analyses on discrete single meta-omics, or even better, microbiome multi-omics datasets, can provide additional and complementary information to explore such diverse communities [196]. On the other hand, progress may be further accelerated by the rise of single-cell multi-omics technologies that offer a single-cell resolution insight into host molecular characteristics in a way that bulk -omics cannot [197]. Ideally, despite undeniable practical, financial and computational obstacles, incorporating different types of multi-omics data from both the host and host microbiota could revolutionize the understanding of diseases as complex as asthma [198].

Such developments will hopefully establish microbial signatures that may serve as key prognostic or diagnostic predictors of asthma phenotypes/endotypes. In COPD for example, the presence of *Staphylococcus* and absence of *Veillonella* in the sputum during exacerbations has been shown to predict future mortality risk [199]. In addition, a global knowledge of how specific microbial species can influence host immunity and physiology would allow designing novel microbiome-based personalized strategies for asthma prevention, therapy or enhanced response to conventional treatments. To this end, modification of the microbiome through nutritional interventions, vaccines, probiotics, bioactive compounds (such as SCFA) supplementation or microbiota transplants may provide new prospects for future investigation. For example, it has been shown that reversing microbiome changes by oral administration of certain *Lactobacillus* and *Bifidobacterium* species may offer some protection against both the initial development of allergy and further exacerbations of atopic disease [200].

## 10. Conclusions

A large scientific effort to a systems biology-oriented stratification of asthma has been recently accomplished, resulting in a deeper molecular characterization and more personalized treatment options for persistent Th2-high asthma. However, a comprehensive definition of severe non-Th2 endotypes—such as neutrophilic asthma—and their targeted management remains a priority.

Extensive airway microbiome studies on clinically characterized cohorts of asthmatics have succeeded in the delineation of associations between microbial signatures and specific disease phenotype/endotypes or health status and shed light on microbiota-treatment relationships. Integration of large-scale metagenomics and host multi-omics big data is currently becoming a priority toward a rapid evolution of knowledge about host–microbiota interactions, new multivariate prognostic/diagnostic biomarkers and potential therapeutic targets. Such thorough understanding would ultimately advance personalized therapies tailored to prevent or treat specific microbial dysbiosis and host immune dysfunction, hopefully leading to improved outcomes for individuals with uncontrolled types of asthma.

## Figures and Tables

**Table 1:** Overview of studies analyzing the airway bacterial microbiome association to asthma inflammatory endotypes.

Year	Participants	Sample Type	Key Findings	Reference
2014	-28 severe asthmatics	Sputum	-Neutrophilic asthmatics: ↑ abundance of pathogenic bacterial species (*Haemophilus* sp., *Streptococcus* sp., *Moraxella catarrhalis*)	[117]
2015	-40 severe asthmatics	Bronchial (Brushings)	-Eosinophils: negative correlation with relative abundance of *Proteobacteria* (*Moraxellaceae, Helicobacteraceae* families), positive correlation with *Actinobacteria* (*Streptomyces* and *Propionicimonas* species)	[146]
2016	-30 asthmatics	Sputum	-Neutrophilic vs. non-neutrophilic asthmatics: ↓ evenness and richness of bacterial species, ↑ *Proteobacteria* (*Haemophilus influenzae)* ↓ *Actinobacteria, Firmicutes* -Eosinophilic asthmatics: ↑ abundance of *Actinobacteria* (*Tropheryma whipplei*)	[148]
2016	-26 severe asthmatics -18 non-severe asthmatics -12 healthy controls	Sputum	-Eosinophils: ↑ *Firmicutes* (*Streptococcus* sp.)	[149]
2017	-23 steroid-free asthmatics -10 healthy controls	BAL ^1^	-Eosinophilic asthmatics vs. healthy controls: ↑ *Neisseria, Bacteroides and Rothia* ↓ *Sphingomonas, Halomonas, Aeribacillus* -Neutrophilic asthmatics vs. healthy controls: differences in *Flavobacterium, Phenylobacterium, Brevundimonas, Bradyrhizobium, Sediminibacterium, Gemella*	[150]
2017	-25 severe asthmatics -24 non-severe asthmatics -15 healthy controls	Sputum	-Eosinophilic vs. non-eosinophilic asthmatics: ↑ *Actinomycetaceae, Enterobacteriaceae* family members	[151]
2017	-42 atopic asthmatics -21 atopic non-asthmatics -21 non-atopic healthy controls	Bronchial (Brushings) Oral wash	-T2-high vs. non-Th2: ↓ bronchial bacterial burden	[118]
2018	-20 neutrophilic asthmatics -34 non-neutrophilic asthmatics	Sputum	-Neutrophilic vs. non-neutrophilic asthmatics: ↑ total bacterial burden, ↓ *Firmicutes, Actinobacteria, Saccharibacteria,* ↑ *Bacteroidetes* phyla (*Porphyromonas* spp., *Capnocytophaga* spp.), *Proteobacteria* (*Haemophilus* spp., *Moraxella* spp.)	[152]
2018	-84 eosinophilic asthmatics -14 neutrophilic asthmatics -60 paucigranulocytic asthmatics -9 mixed neutrophilic and eosinophilic asthmatics	Sputum	-Neutrophilic asthmatics vs. all other endotypes: ↓ diversity, richness and evenness, ↑ high relative abundance in pathogenic taxa (*Haemophilus* and *Moraxella*), ↓ *Streptococcus*, *Gemella* and *Porphyromonas* -Eosinophilic vs other endotypes: ↓ *Haemophilus, Gemella, Rothia and Porphyromonas*	[153]
2018	-32 asthmatics -73 COPD ^2^ patients	Sputum	-Neutrophilic asthmatics: ↑ *Proteobacteria* phyla -Eosinophilic asthmatics: ↑ *Bacteroidetes*	[154]
2019	-10 eosinophilic asthmatics -14 non-eosinophilic asthmatics -12 healthy controls	Sputum	-Eosinophilic vs. non-eosinophilic asthmatics: ↑ richness, evenness and diversity, ↑ *Glaciecola*, *Helicobacter* ↓ *Scardovia, Bifidobacterium, Desulfobulbus,* *Deinococcus*	[155]
2020	-32 atopic asthmatics -18 atopic non-asthmatics -16 non-atopic healthy controls	Sputum BAL Oral wash	-T2-high vs. non-Th2: ↓ Sputum bacterial burden	[156]
2021	-100 severe asthmatics	Sputum	-High neutrophilic vs. low neutrophilic asthmatics: ↓ richness and diversity, ↑ increased relative abundance of pathogenic species (*Haemophilus influenzae*, *Moraxella catarrhalis*, *Streptococcus pseudopneumoniae*) ↓ *Veillonella, Prevotella and Neisseria*	[157]

^1^ BAL: bronchoalveolar lavage; ^2^ COPD: chronic obstructive pulmonary disease.

**Table 2:** List of multi-omics integration studies in asthma.

Year	Asthma Type	Sample Types	Multi-Omics Data	Reference
2015	Pediatric vs. Healthy	Nasal (Brushings)	Metatrascriptome Transcriptome (RNA sequencing, in silico separation)	[170]
2015	Pediatric vs. Healthy	Nasal (Brushings)	Metatrascriptome Transcriptome (RNA sequencing, in silico separation)	[171]
2015	Severe vs. Healthy and Mild-to-Moderate	Bronchial (Brushings)	Metagenome (16S rRNA gene array) Transcriptome (Gene expression microarray)	[146]
2020	Severe Persistent Childhood vs. Healthy	Nasal (Brushings-swabs) Bronchial (Brushings-BAL ^1^)	Metagenome (16S rRNA gene sequencing) Transcriptome (RNA sequencing)	[172]
2020	Mite-sensitized Childhood vs. Healthy	Airway (Throat swabs) Blood (Serum)	Metagenome (Shotgun metagenome sequencing) Metabolome (NMR)	[173]
2020	Allergic Childhood Obese vs. Non-obese and Persistent vs. Non-persistent	Fecal (Stool) Blood (Plasma)	Metagenome (16S rRNA gene sequencing) Metabolome (NMR and GC-MS) Proteome (Luminex and ELISA)	[174]
2021	Severe vs. Healthy and Mild-to-Moderate	Sputum	Metagenome (16S rRNA gene sequencing) Transcriptome (Gene expression microarray) Proteome (SomaScan assay proteomics)	[178]
2021	Severe vs. Healthy and Mild-to-Moderate	Nasal (Brushings)	Metagenome (16S rRNA gene sequencing) Transcriptome (RNA sequencing) Methylome (Infinium MethylationEPIC array)	[179]

^1^ BAL: bronchoalveolar lavage.
